# Creating positive learning experiences with technology: A field study on the effects of user experience for digital concept mapping

**DOI:** 10.1016/j.heliyon.2022.e09246

**Published:** 2022-04-09

**Authors:** Björn Rohles, Susanne Backes, Antoine Fischbach, Franck Amadieu, Vincent Koenig

**Affiliations:** a Cognitive Science & Assessment, Faculty of Humanities, Education and Social Sciences (FHSE), University of Luxembourg, 2, avenue de l'Université, L-4365 Esch-sur-Alzette Luxembourg; b Luxembourg Centre for Educational Testing (LUCET), University of Luxembourg, 2, avenue de l'Université, L-4365 Esch-sur-Alzette, Luxembourg; c CLLE (CNRS-UT2J), University of Toulouse, Laboratoire CLLE UMR5263, Université Toulouse Jean Jaurès, Maison de la Recherche, 5, Allée Antonio Machado, 31058 Toulouse cedex 9, France

**Keywords:** User experience, Psychological needs, Intention to use, Concept mapping, UX, User experience

## Abstract

Learning and assessment are increasingly mediated by digital technologies. Thus, learners’ experiences with these digital technologies are growing in importance, as they might affect learning and assessment. The present paper explores the impact of user experience on digital concept mapping. It builds on user experience theory to explain variance in the intention to use digital concept mapping tools and in concept map-based assessment scores. Furthermore, it identifies fulfillment of psychological needs as an important driver of positive experiences. In a field study in three schools and a university (N = 71), we tested two concept mapping prototypes on computers and tablets. We found that user experience is a significant factor explaining variance in intention to use. User experience also explained variance in three out of four concept mapping scores on tablets, potentially related to the lower pragmatic quality of the tablet prototypes. Fulfillment of psychological needs strongly affected perceptions of different qualities of user experience with digital concept mapping. These results indicate that user experience needs to be considered in digital concept mapping to provide a positive and successful environment for learning and assessment. Finally, we discuss implications for designers of digital learning and assessment tools.

## Highlights

• User experience (UX) is a vital success factor for digital education tools.
• Fulfillment of psychological needs defines user experience.
• User experience is a strong predictor of intention to use.
• User experience can explain variance in concept map scores.
• User experience models can complement technology acceptance models.

## 1 Introduction

The field of education is increasingly addressing “21st century digital skills” like critical thinking and problem-solving (van Laar et al., 2017). Concept mapping is a promising method for acquiring these skills (Novak, 2010). Furthermore, increasing attention is being paid to the subjective experiences that shape learning, like engagement, interests, motivation, or needs (Norman and Spohrer, 1996; Reigeluth et al., 2017), leading to a greater focus on the learner. For instance, the fulfillment of psychological needs has been found to impact learning and assessment (Evans et al., 2012; Masters et al., 1979; Pekrun, 2006; Tien et al., 2018). When it comes to designing digital tools for education, the concept of “user experience” has emerged to capture such subjective experiences with technology (Bargas-Avila and Hornbæk, 2011).

The purpose of this paper is to empirically investigate the role of user experience (UX) in digital concept mapping, particularly its relationship with psychological needs, intention to use, and scores on a concept map-based assessment. In this way, the paper contributes to establishing UX as a phenomenon of interest in research on digital education, particularly with respect to the role of UX in knowledge assessment. Such assessments can be a high-stakes operation for learners, because scores could directly impact their future educational trajectories. Thus, it is vital to ensure that UX aspects do not influence learners’ future opportunities.

The novelty of this paper is the broader picture that we draw by incorporating antecedents (i.e., psychological need fulfillment) and outcomes (intention to use, scores) to investigate the role of user experience in digital education. Specifically, we build on digital concept mapping as a case study. To the best of our knowledge, no prior study has investigated user experience in digital concept mapping in a similar approach. In the following section, we will outline these considerations in detail.

## 2 Current state of research

**Figure 1:**
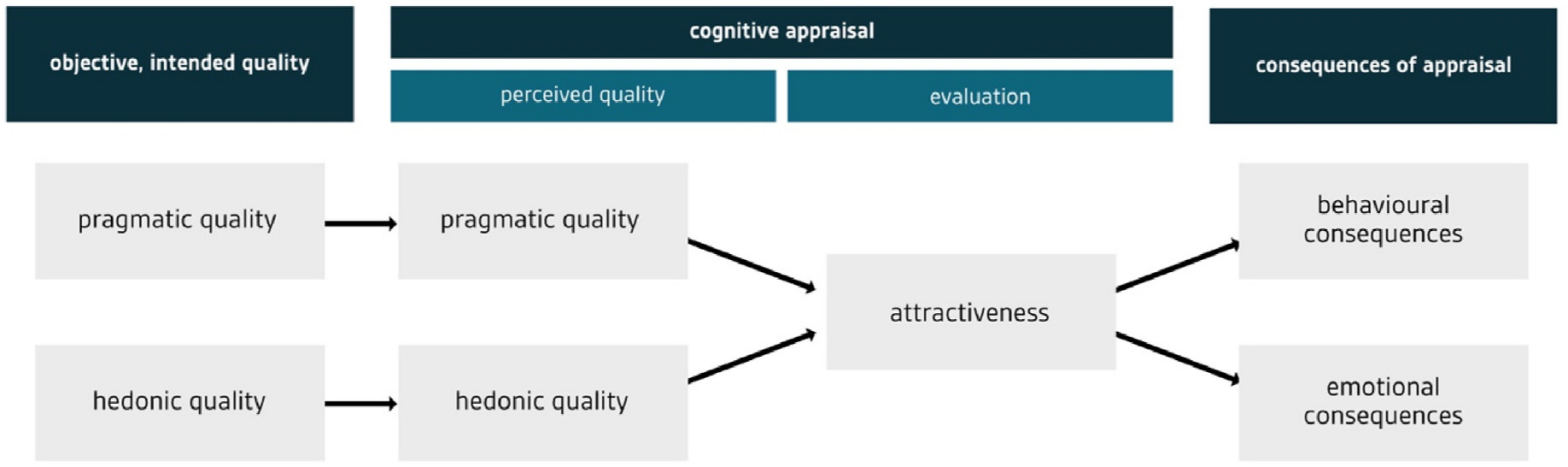
Model of user experience, adapted from Hassenzahl (2001). Based on objective pragmatic and hedonic qualities of a product, users form a perceived impression of the pragmatic and hedonic qualities. Those contribute to users' evaluation of attractiveness. Attractiveness is assumed to influence consequences, such as behavior and emotions.

In the last two decades, the impact of technology on human beings has been increasingly discussed from a user experience (UX) perspective. There are many models of UX, such as the one suggested by Hassenzahl (2001) (see Figure 1). This model provides a good foundation for investigating the role of UX in assessment because it includes consequences of UX and outlines how characteristics of a product (e.g., a digital education technology) relate to characteristics of the experience. According to this model, digital products have pragmatic and hedonic qualities. When users interact with a product, they build a subjective impression of these pragmatic and hedonic qualities, and these are referred to as the pragmatic and hedonic dimensions of UX. The pragmatic dimension refers to instrumental or ergonomic aspects, termed the “do-goals” of interaction, and covers usability components like ease of use or efficiency (Hassenzahl, 2008). The hedonic dimension refers to the fulfillment of deeper psychological needs (like feeling competent or feeling stimulated), known as “be-goals” (Hassenzahl, 2008). Users form an overall judgement of the attractiveness of the product based on their impressions of its quality. This judgement has consequences for their behavior (e.g., use of the technology) and experience (e.g., emotional reactions). Recent research explores whether so-called eudaimonic quality constitutes a third dimension of user experience (Mekler & Hornbæk, 2016, 2019). Eudaimonic quality refers to the development of one’s full potential (Huta and Ryan, 2010) and is thus particularly important in the field of education.

In the following sections, we will first examine research on antecedents of UX in digital education, namely psychological needs, before turning to the outcomes of UX in digital education, namely intention to use and learning success.

### 2.1 Psychological needs as drivers of experience

Psychological needs play an important role in creating positive experiences (Hassenzahl et al., 2010; Tay and Diener, 2011). Psychological needs are defined as “basic requirements for the functioning of an organism” (Desmet and Fokkinga, 2020, p. 2) and are a substantial source of motivation (Levesque et al., 2004). Several theories have been developed that identify specific psychological needs among humans. For example, Abraham H. Maslow assumed a hierarchical ordering of human needs (Maslow, 1943). However, empirical research does not support the notion of a universal hierarchical ordering of needs (Desmet and Fokkinga, 2020). Self-determination theory (Deci and Ryan, 2000; Orkibi and Ronen, 2017), which identified autonomy, competence, and relatedness as basic psychological needs, has received support from a range of studies (Levesque et al., 2004; Oostdam et al., 2018). Sheldon et al. (2001) synthesized research on human needs into a list of ten basic needs: self-esteem, autonomy, competence, relatedness, pleasure/stimulation, physical thriving, self-actualizing/meaning, security, popularity/influence, and money/luxury. A range of design methods for needs fulfillment have been developed on the basis of this synthesis of needs (Diefenbach et al., 2014; Desmet and Fokkinga, 2020; Lallemand, 2015).

Numerous studies have demonstrated the importance of needs in learning and assessment, e.g., in the context of physical education (Katartzi and Vlachopoulos, 2011), learning to play a musical instrument (Evans et al., 2012), avoiding maladaptive behaviors in school (Oostdam et al., 2018), or promoting psychological well-being (Orkibi and Ronen, 2017; Tay and Diener, 2011). Needs are important for experiencing an activity as pleasurable and meaningful (Desmet and Fokkinga, 2020). These positive emotions can significantly enhance learning and assessment (Masters et al., 1979; Tien et al., 2018; Yang et al., 2013) in areas such as creativity tasks (Fredrickson, 1998; Isen et al., 1985; Lyubomirsky et al., 2005) and decision-taking tasks (Carpenter et al., 2013). Interest in psychological needs in design has recently been growing, particularly regarding how to design for motivation, engagement, and well-being through fulfillment of psychological needs (Peters et al., 2018; Wannheden et al., 2021). Thus, identifying how digital education tools contribute to such need fulfillment is vital when education is increasingly digitalized.

### 2.2 Intention to use

The success of digital products depends on users’ willingness to use them. The question of which aspects determine whether a person will adopt a given technology has been addressed from various angles (Alexandre et al., 2018), such as the technology acceptance model (TAM) tradition (Davis et al., 1989; Venkatesh and Davis, 2000; Venkatesh, 2000; Venkatesh and Bala, 2008). In line with the growing recognition of the value of experience (Newman and Blanchard, 2016; Pine and Gilmore, 1998; Solis, 2015), research on technology adoption increasingly addresses the UX perspective (Alexandre et al., 2018; Hornbæk and Hertzum, 2017). Although the boundaries and relations between UX-based acceptance theories and other theories like TAM are not always clear (Hornbæk and Hertzum, 2017; Alexandre et al., 2018), it is generally agreed that UX can contribute to explaining what drives intention to use a technology. There are two reasons for this. First, the strong rooting of UX in psychological factors such as the aforementioned psychological needs helps to clarify how these factors contribute to technology adoption (Alexandre et al., 2018; Hornbæk and Hertzum, 2017). UX models like the one by Hassenzahl (2001) are rarely used to predict experiences and outcomes (Hornbæk and Hertzum, 2017), but exploring such alternative models of intention to use has been encouraged (Šumak et al., 2011). Second, UX places a strong emphasis on the role of different dimensions of experience, such as the pragmatic and hedonic dimensions (Hassenzahl, 2008), for technology adoption. Thus, a UX-based perspective on intention to use could provide recommendations for designing digital products and services that are likely to be adopted by their envisioned users (Hornbæk and Hertzum, 2017). Recently, researchers have started to include experience into technology acceptance models (Ahmad et al., 2021; McLean et al., 2018). This paper contributes to this research by providing a perspective grounded in UX theory: It provides a broad picture of the impacts of experience by incorporating antecedents (i.e., psychological need fulfillment) and outcomes (i.e., scores in assessment, see following) in the analysis.

### 2.3 User experience in learning and assessment

Research on the impact of user experience on learning and assessment has often focused on pragmatic aspects, especially usability. Usability impacts learning success (Tselios et al., 2008) and assessment (Tselios et al., 2001; Weinerth et al., 2013). This effect is frequently discussed in terms of cognitive load theory, with the suggestion that it reduces so-called extraneous cognitive load (due to design aspects such as usability issues) so that learners can invest their mental resources in task-relevant activities (Amadieu et al., 2015; Hollender et al., 2010; Sweller, 2010).

Although UX includes these pragmatic considerations, the concept of UX also encompasses the hedonic dimension. Hedonic aspects like joy and motivation are also likely to influence learning and assessment (Hollender et al., 2010). For example, positive emotions enhance learning (Masters et al., 1979; Tien et al., 2018) and heighten learners’ willingness to invest mental resources in learning (Efklides et al., 2006). Numerous studies found relations of UX and learning, for example with serious games (Espinosa-Curiel et al., 2020). Thus, digital learning and assessment tools should impact learning not only from the perspective of usability and cognitive load, but also by creating a positive, engaging environment.

The present study focuses on digital concept mapping as a case study for investigating the role of UX. Concept mapping (Novak and Gowin, 1984) is a method of visually representing relationships within complex knowledge. It consists of concepts (in the form of shapes) connected by labelled links (arrows). Meaningful units of at least two concepts and links are known as propositions (Shavelson et al., 2005). Concept mapping is a very promising case study for evaluating the role of UX in digital education for several reasons. First, only a few studies systematically investigate UX in concept mapping (e.g., see Weinerth et al., 2014 for usability in concept mapping). Recently, Pinandito et al. (2020) investigated acceptance of a particular concept mapping system (called Kit-Build) in the framework of technology acceptance. They found that perceived enjoyment and ease of use significantly predict students’ evaluations of a systems’ usefulness, providing evidence to the importance of UX in concept mapping. However, no holistic exploration of the role UX plays in concept mapping has been conducted. Second, concept mapping is a promising approach for addressing contemporary challenges in education like systems thinking (Assaraf and Orion, 2005; Brandstädter et al., 2012; Cox et al., 2019) or assessing complex knowledge structures (Shavelson et al., 2005). As cognitive structures are not directly observable, concept mapping allows learners to create a visualization of their understanding of a topic (Ifenthaler, 2010). Concept maps can be assessed with respect to a variety of different aspects, such as their comprehensiveness, organization, or correctness (Besterfield-Sacre et al., 2004). Third, concept mapping is a complex task where learners need to constantly elaborate and reflect on the propositions they create (Amadieu et al., 2009; Sanchiz et al., 2019). Digital concept mapping tools have the potential to facilitate these processes (Hwang et al., 2012) and enable the creation of more complex concept maps that better reflect learners’ knowledge (Brandstädter et al., 2012). However, these potential benefits depend on the qualities of the digital concept mapping tool itself, or in other words, how users experience it.

### 2.4 Research questions

The purpose of this paper is to examine how user experience impacts digital concept mapping. In particular, it addresses the relations among UX, fulfillment of psychological needs, intention to use, and assessment scores for digital concept mapping. The research questions (RQ) were defined as follows: How do psychological needs affect UX in digital concept mapping (RQ 1)? How does UX affect intention to use digital concept mapping (RQ 2)? How does UX affect scores in digital concept mapping (RQ 3)?

In line with the role of psychological needs in creating positive experiences, we hypothesized that need fulfillment would significantly predict the pragmatic and hedonic dimensions of UX:

Hypothesis 1 The fulfillment of psychological needs significantly predicts the pragmatic and hedonic dimensions of UX.

Furthermore, we hypothesized that attractiveness would explain variance in intention to use and concept mapping scores. With respect to intention to use, we assumed that learners would be more willing to use a digital tool with a higher UX because it allows them to reach their pragmatic and hedonic goals. With respect to concept mapping scores, we assumed that a digital tool with a higher UX would lead to reduced extraneous cognitive load (pragmatic aspects; Sweller, 1994; Sweller et al., 2011) and enhanced motivation (hedonic aspects). These advantages would allow learners to create higher-quality concept maps:

Hypothesis 2 Attractiveness significantly predicts intention to use.

Hypothesis 3 Attractiveness significantly predicts concept mapping scores.

**Figure 2:**
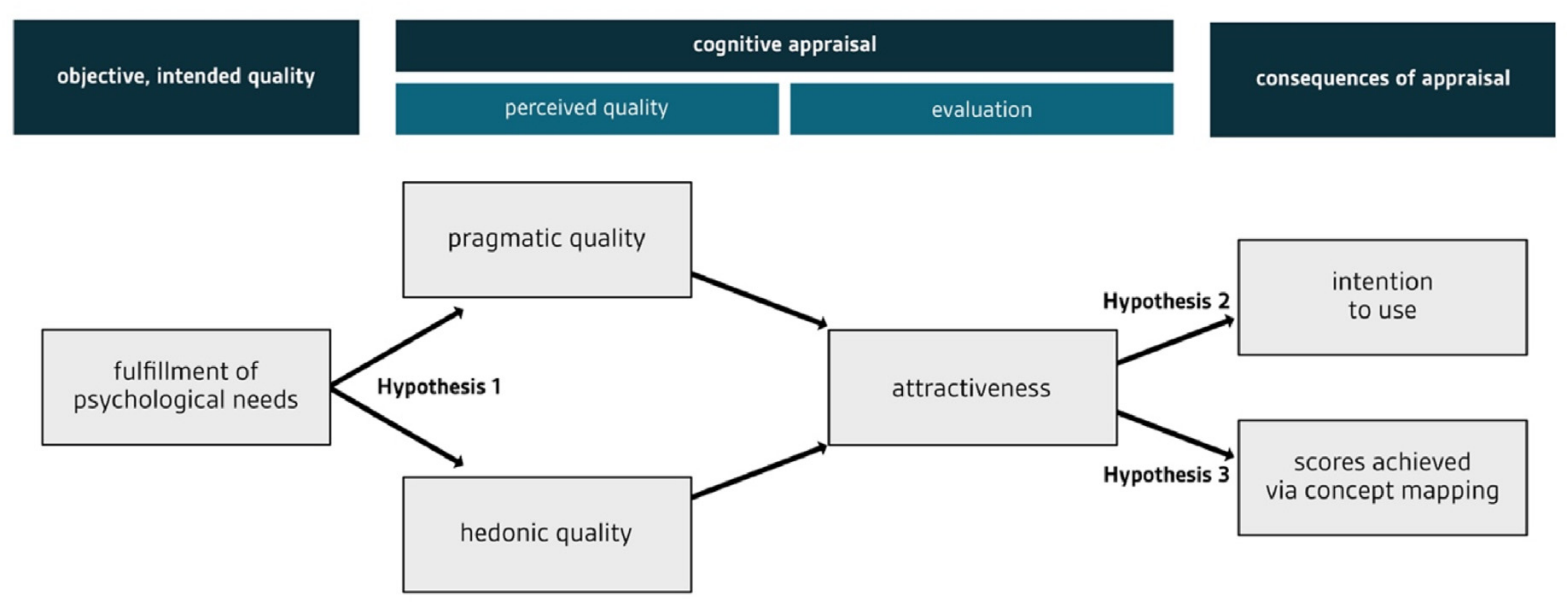
Hypotheses of the present study, adapted from Hassenzahl (2001). Hypothesis 1 checks whether fulfillment of psychological needs explains variance in perceived pragmatic quality and perceived hedonic quality. Hypothesis 2 checks whether attractiveness explains variance in intention to use. Hypothesis 3 checks whether attractiveness explains variance in concept map scores.

An overview of the hypotheses is represented in Figure 2.

## 3 Materials and methods

We aimed to examine an ecologically valid context and conducted a field experiment in educational institutions across Luxembourg. Four classes in three different schools and a group of university students participated in the study (*N* =71^1^, see Table 1).

**Table 1:** Participants and settings of the study.

School	Grade	Age	N	Setting
Secondary school – technical track	3e (11^th^ grade)	*M* = 17.20 yr; *SD* = 1.612 yr	10 male 5 female	Computer, class
Secondary school – academic track	1e (13^th^ grade)	*M* = 18.29 yr; *SD* = 0.463 yr	7 male 14 female 1 no data	Tablet, class
Secondary school – academic track	4e (10^th^ grade)	*M* = 15.50 yr; *SD* = 0.535 yr	6 male 2 female	Tablet, remote
Secondary school – academic track	4e (10^th^ grade)	*M* = 15.50 yr; *SD* = 0.699 yr	3 male 7 female	Tablet, remote
University	Bachelor: 10 Master: 4 PhD: 2	*M* = 23.19 yr; *SD* = 4.339 yr	8 male 8 female	Computer, class: 9 Tablet, class: 3 Computer, remote: 3 Tablet, remote: 1

**Figure 3:**
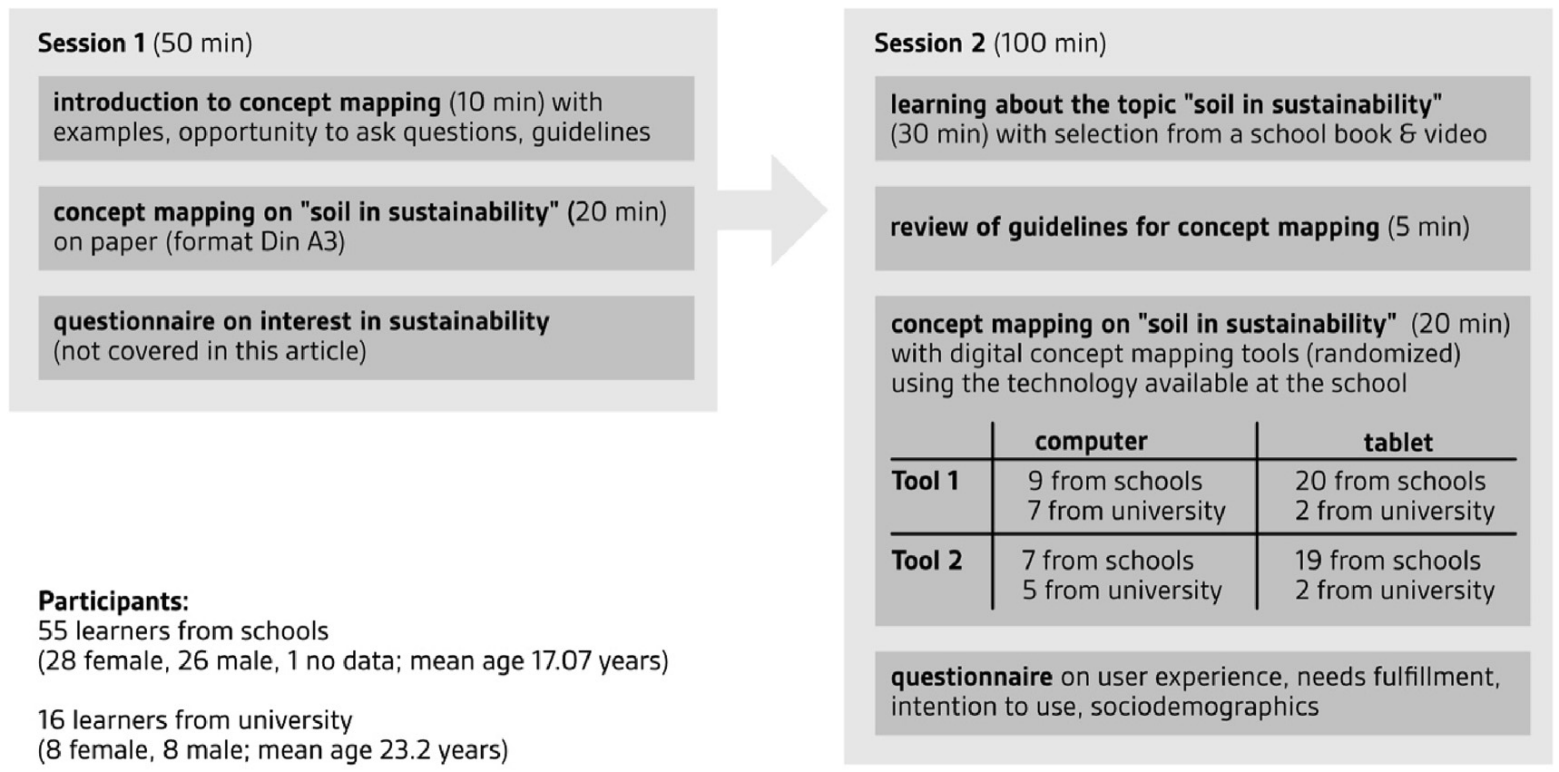
Setup of the study. Study with 55 learners from schools (28 female, 26 male; mean age 17.07 years) and 16 learners from university (8 female, 8 male, mean age 23.2 years). In Session 1 (50 minutes), learners received an introduction to concept mapping, created a concept map on soil in sustainability, and answered a questionnaire on their interests in sustainability. In session 2 (100 minutes), they learned about the topic, reviewed guidelines for concept mapping, and created a second concept map. This time, they used one of two tools on a technology available at the schools. The numbers were: Tool 1 on computers (9 from school, 7 from university) and tablets (20 from school, 2 from university); Tool 2 on computers (7 from schools, 5 from university) and tablets (19 from schools, 2 from university). Finally, they ansered a questionnaire on user experience, needs, intention to use and sociodemographics.

The study was part of a research project investigating the role of UX in digital concept mapping. Previous studies in this project investigated the target audiences of digital concept mapping in education. We identified a strong need for the method (and an appropriate tool) in secondary and tertiary education, making our sample of students from schools and universities representative of the audience we expect. The classes were specifically recruited to represent different tracks and grades to represent the diversity of the student population in Luxembourg. The project obtained ethical approval from the Ethics Review Panel of the University of Luxembourg (ERP, 18031). Strict ethical guidelines of informed consent by participants and (for minors) their parents were applied. Participants received compensation of € 50 (university participants) or were released from regular instruction for participation (school participants). The study took place in two sessions, usually at an interval of 3–7 days. Materials from the sessions were linked using a subject-generated identification code. The identification codes mentioned in this article were re-coded to safeguard anonymity. Figure 3 provides an overview of the study setup.

### 3.1 Pre-tests and study setting

Our study setup originally intended to incorporate digital concept mapping in both sessions. All procedures and materials were pre-tested with a class of 7 learners (6 male, 1 female, mean age 17.4 years). The pre-test revealed that not all schools were able to provide digital devices for two sessions. Furthermore, the learners had difficulties learning both the method and a particular concept mapping tool simultaneously. Consequently, we conducted paper-based concept mapping for the first session as a baseline measurement.

In Session 1, learners received a standardized introduction to concept mapping with guidelines (Novak, 2010) and created a paper-based concept map of their prior knowledge on the topic of “soil in sustainability” (about 20 min). In Session 2 (100 min), participants learned about the study topic “soil in sustainability” (30 min) and created a digital concept map (about 20 min). This topic was chosen because we expected a low level of systematic prior knowledge, as this topic is rarely discussed in detail in the media or the school curriculum. The learning material consisted of a selection from a school textbook (Hoffmann, 2018) and a video (Streckenbach, 2012) by the renowned German Institute for Advanced Sustainability Studies (IASS). These materials were chosen for their quality (as verified by an external expert and via specific questions in the questionnaire) as well as their use of different input modalities (visual and verbal information) to support different processes in working memory (Baddeley, 2000). All materials were available in the three most important languages in the multilingual country in which the study took place. After the learning phase, the concept mapping guidelines were reviewed and remained available to learners during the concept mapping activity (about 20 min). Finally, data on UX, need fulfillment, sociodemographic background, and intention to use was collected with a questionnaire (described in Section 3.4).

As the study intended to achieve high ecological validity, learners participated with the technologies available at their schools (tablets or computers) and in the same teaching conditions as regular instruction (in-person in the classroom or remotely during Covid-19 lockdowns). Care was taken to keep the study conditions identical by strictly standardizing all procedures (e.g., with the help of video recordings for remote participants). Furthermore, we performed a manipulation check to verify which contextual factors impacted UX (described in Section 3.5).

To achieve our aim of conducting the study in a setting with high ecological validity, we randomly assigned participants to one of two fully functional concept mapping tools. The two tools were developed to focus on different points along the continuum of pragmatic and hedonic characteristics (Hornbæk and Hertzum, 2017): Tool 1 was optimized primarily for pragmatic UX, while Tool 2 was optimized for both pragmatic and hedonic UX. However, as we will outline in Section 3.4, the prototypes did not create systematically different UX.

### 3.2 Description of prototypes

**Figure 4:**
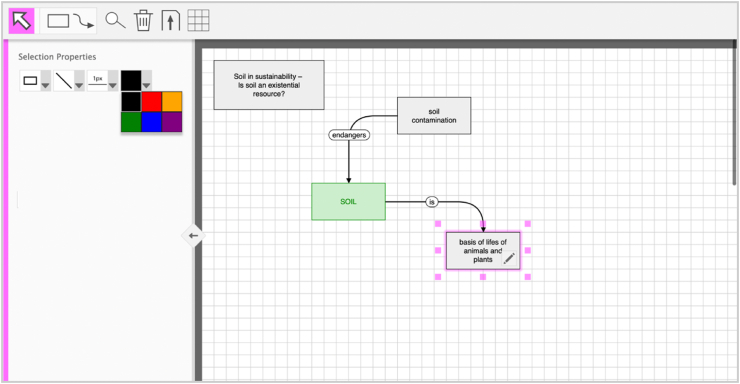
Tool version 1 focused on optimizing for usability. Interface of tool 1 with basic editing options.

Tool 1 (see Figure 4) is based on design suggestions for digital concept mapping tools from a project focused on optimizing for pragmatic UX in digital concept mapping, particularly with respect to usability (Weinerth, 2015). The suggestions were derived following a user-centered design process in three iterations with 90 user tests. Tool 1 implements the derived suggestions. In particular, it has the following features:

• basic shapes (four shapes, with dedicated modes for creating one or unlimited objects),
• links that automatically connect to objects (and update when the objects are moved),
• limited options for styling elements (six colors, six line types, four line thicknesses)
• dedicated zoom and scroll modes on tablets (when activated, touching the screen adapts the canvas display rather than creating new elements).

**Figure 5:**
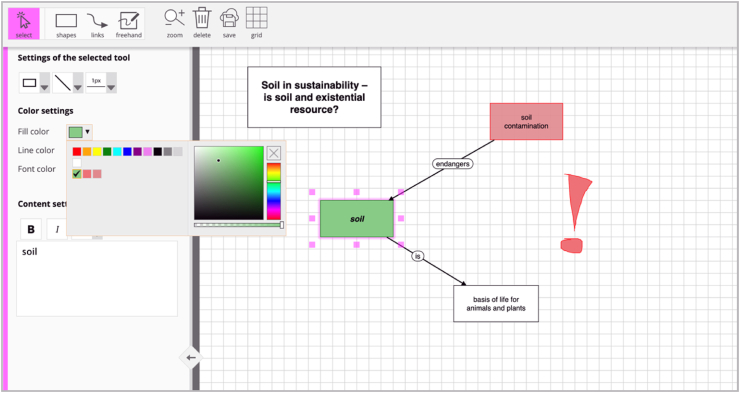
Tool 2 with stronger focus on holistic user experience. Interface of tool 2 with advanced editing options.

Tool 2 (see Figure 5) was developed in a project focused on optimizing for holistic UX, and thus including both the pragmatic and hedonic dimensions. It was based on user research (with a total of 88 participants) and two iterations of user tests (with a total of 66 participants). Two main changes were instituted compared to Tool 1. First, Tool 2 addressed a series of usability issues discovered in the tests, specifically by removing the option to switch between modes for creating single or unlimited objects, enhancing the menu icons by including labels, and providing onboarding instructions at the beginning that explain the most important tools. Second, Tool 2 includes a set of hedonic options frequently desired by users, specifically enhanced styling options and a basic freehand drawing tool for adding manual annotations (like exclamation marks).

### 3.3 Concept mapping

In this study, we elected to provide participants with a list of suggested terms and a focus question (“Soil – an existential resource?”) to improve the accuracy of participants’ propositions (Brandstädter et al., 2012; Ruiz-Primo, 2004). This list of 31 concepts and ten links was extracted from a reference concept map created by an independent domain expert. Learners were allowed to add their own terms. Furthermore, we included two distractors that were not necessary to describe the topic (Strautmane, 2012).

The quality of participants’ concept maps was scored with a scoring rubric developed by Besterfield-Sacre et al. (2004) and a reference concept map reflecting expert knowledge of the topic. Rubrics describe criteria and levels of performance and are well-established in education (Brookhart, 2013). The rubric by Besterfield-Sacre et al. (2004) evaluates three dimensions: comprehensiveness (how well the concept map explains the topic), organization (how interconnected the concept map is), and correctness (whether the concept map contains misconceptions). Previous research has successfully applied the rubric to studying sustainability (Watson et al., 2016). The original scoring rubric was adapted to the topic of “soil in sustainability” and converted into a scale by allowing mid-values (e.g., between 1 and 2) (see Table 2). Furthermore, we gave concept maps that completely failed to describe the topic a score of 0.

**Table 2:** Scoring rubric for evaluating the concept maps based on Besterfield-Sacre et al. (2004).

	0	1	2	3
Comprehensiveness	The map does not define the topic or is completely off-topic. The knowledge is not visible or not related to the topic.	The map lacks an adequate definition of its subject (for example, no central concept visible or central concept too general). The knowledge is very simple and limited. Low breadth of concepts (for example, relevant aspects are only minimally covered, no or limited mentioning of important sustainability categories). The map barely covers the topic.	The map defines the topic adequately (for example by defining a relevant central concept or a focus question). However, the knowledge is limited in some areas (for example, some key areas of sustainability and relevant aspects are covered but others are missing). The map demonstrates a limited understanding of the topic (for example because relations and dependencies within the area of sustainability are only covered to a limited extent).	The map completely defines the topic. Regarding content, only a few aspects of sustainability are missing (for example, all relevant categories of sustainability and numerous content areas are covered, like ecological, economic, and social factors).
Organization	The concepts in the map are not at all or mostly not connected. There are no visible branches or other structures in the concept map.	The concepts in the map are only linearly connected. There are only a few or no connections between branches of the map. Concepts are not well integrated.	The map has an adequate organization within some branches. Some signs of integrating different areas are visible, but not completely. Some feedback loops or other dependencies are depicted.	The map is well organized and captures several feedback loops or other dependencies. The structure is highly developed and well connected.
Correctness	The correctness of the map cannot be evaluated. Numerous concepts are unlabeled or not readable.	The map is simplistic and contains numerous misconceptions about the topic. Inappropriate terms are used. The map reflects an inaccurate understanding of the topic.	The map has some misconceptions about the topic. However, most relations are correct. There are some smaller errors and incorrect relations concerning the field of sustainability.	The map integrates the concepts very well and demonstrates a thorough understanding of the topic. There are few or no misconceptions or other errors. The central relations within the field of sustainability are covered.

Participants’ paper concept maps (Session 1) and digital concept maps (Session 2) were independently analyzed by two researchers. The two researchers then discussed their ratings and reached agreement. These scores were then summed up to calculate a global score (Watson et al., 2016). Finally, we subtracted the scores from Session 1 from the scores from Session 2 to arrive at a score reflecting the difference between the concept maps created on paper (independent of any instruction and user experience) and digitally created concept maps (reflecting newly acquired knowledge from the instruction and potential influences of user experience).

### 3.4 Measurements

We applied the User Experience Questionnaire (UEQ; Laugwitz et al., 2008) to measure UX. The UEQ is based on the model of user experience by Hassenzahl (2001) but seeks to capture a balance of pragmatic and hedonic aspects (Laugwitz et al., 2008). Thus, the UEQ is a good fit for our research questions, because both pragmatic and hedonic aspects are important for digital concept mapping tools (Rohles et al., 2019). The UEQ consists of three subscales for pragmatic quality and two subscales for hedonic quality, with four items each. Furthermore, it includes six items measuring the overall attractiveness of the product. Attractiveness is assumed to depend on the ratings on the pragmatic and hedonic scales (Laugwitz et al., 2008). In addition to the UEQ, we included an open question asking for participants’ feedback and ideas regarding the concept mapping tool.

We applied a scale developed by Lallemand and Koenig (2017) based on Sheldon et al. (2001) to measure need fulfillment (see Table 3). In addition, each learner evaluated the importance of each of the seven needs for concept mapping on a 5-point Likert scale (Lallemand and Koenig, 2017).

**Table 3:** Overview of psychological needs used in the present study (Sheldon et al., 2001, p. 339; Lallemand and Koenig, 2017).

Need	Definition	Example item: During this interaction, I felt…
Autonomy and independence	“Feeling like you are the cause of your own actions rather than feeling that external forces or pressures are the cause of your actions”.	… that my actions were based on my interests.
Competence and effectiveness	“Feeling very capable and effective in your actions rather than feeling incompetent or ineffective”.	… that I was successfully completing tasks.
Relatedness and belongingness	“Feeling that you have regular intimate contact with people who care about you rather than feeling lonely and uncared for.”	… a sense of contact with other people in general.
Pleasure and stimulation	“Feeling that you get plenty of enjoyment and pleasure rather than feeling bored and understimulated by life.”	… that I was experiencing new activities.
Security and control	“Feeling safe and in control of your life rather than feeling uncertain and threatened by your circumstances.”	… that things were structured and predictable.
Popularity and influence	“Feeling that you are liked, respected, and have influence over others rather than feeling like a person whose advice and opinions nobody is interested in.”	… that I was a person whose opinion counts for others.
Self-actualizing and meaning	“Feeling that you are developing your best potentials and making life meaningful rather than feeling stagnant and that life does not have much meaning.”	… my actions were with purpose.

Finally, we captured intention to use with a single item “I would use the tool if it were available” on a 5-point Likert scale. Although research often suggested using multiple items (Diamantopoulos et al., 2012) for measuring constructs, an ongoing debate concerns single items for so-called “double concrete” constructs (Bergkvist and Rossiter, 2007; Rossiter, 2002, 2011). These constructs are supposed to have a clear meaning and are unambiguous for the study participants, for example intentions towards behavior (Ang and Eisend, 2018). A recent meta-analysis found no difference in effect sizes between single items and multiple items for these constructs (Ang and Eisend, 2018). Furthermore, our pre-testing revealed no indication of ambiguities or uncertainties for this item from our participants. Finally, the constrained school context did impose strict time constraints: We had to carefully balance data collection with other study requirements (such as learning intervention and debriefing) to ensure that the entire study could take place within regular school lessons. Thus, we opted for using a single item for intention to use in this study but will return to this question in the limitations section. The measurement instruments are available in Appendix.

Participants in the in-person sessions answered on paper, participants in the remote sessions answered via an online survey. All statistical analyses were performed using SPSS version 27. All reported confidence intervals (95% bias-corrected and accelerated) and standard errors are based on 1,000 bootstrapped samples. All significance tests used *p* = 0.05. Significant results are bolded in the tables in this paper.

We calculated reliability of the standardized scales (UEQ and need fulfillment) using McDonald’s ω (Danner et al., 2016; Hayes and Coutts, 2020; McDonald, 1999). ω was calculated with the SPSS macro available from Hayes and Coutts (2020). Reliability of the UEQ items was very high: pragmatic quality (ω = 0.87), hedonic quality (ω = 0.89), and attractiveness (ω = 0.90). Reliability of the need fulfillment was as follows: autonomy and independence (ω = 0.70), competence and effectiveness (ω = 0.85), relatedness and belongingness (ω = 0.77), pleasure and stimulation (ω = 0.76), security and control (ω = 0.74), influence and popularity (ω = 0.61), self-actualizing and meaning (ω = 0.68). We consider most of these values acceptable and return to the remaining issues in Section 4.3.3 when we discuss the study’s limitations.

### 3.5 Manipulation success check of user experience scores

**Figure 6:**
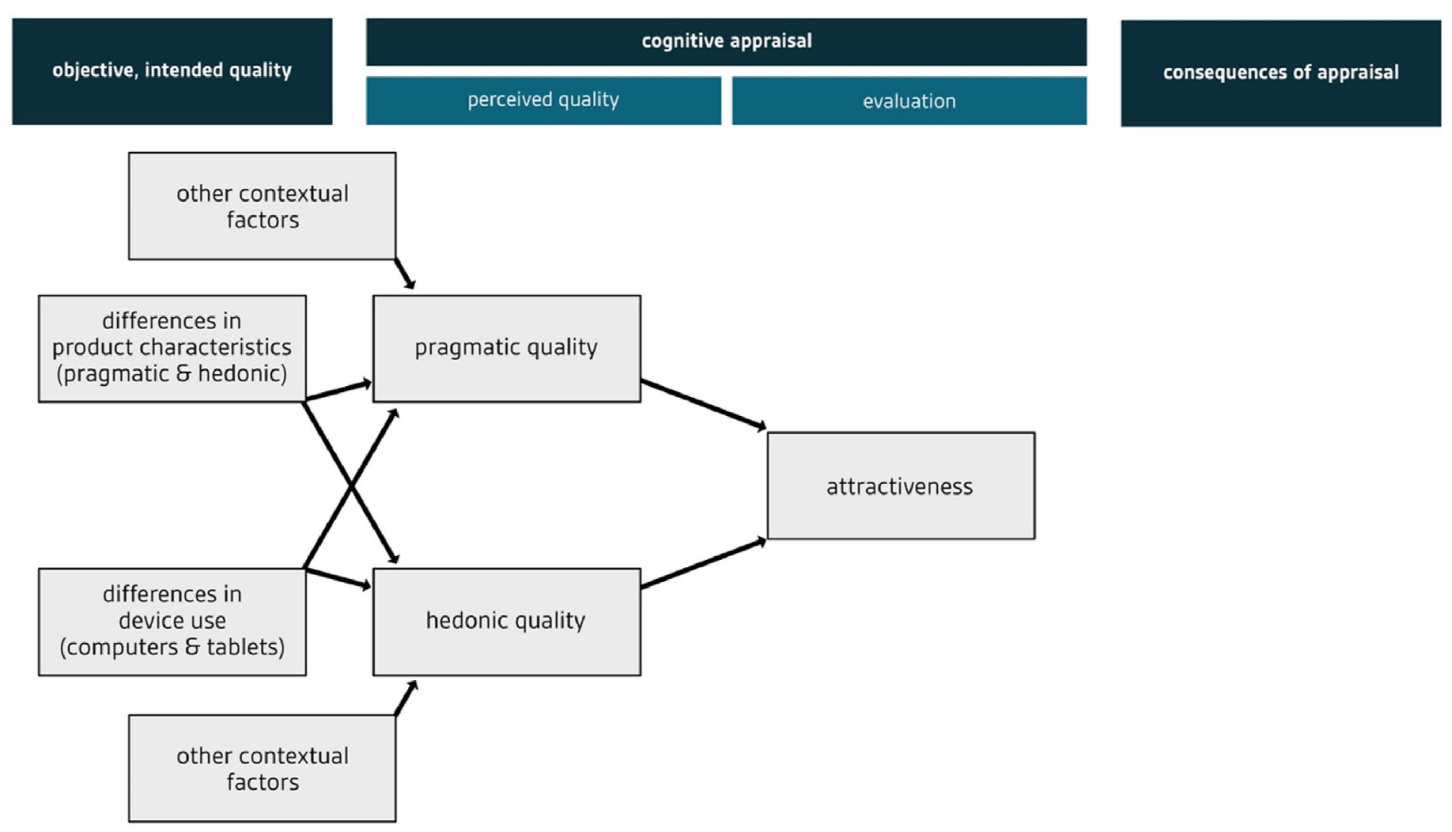
Manipulation checks for the present study, adapted from Hassenzahl (2001). Checks were performed to verify whether differences in settings explained variance in perceived pragmatic and hedonic quality. In detail, we checked for differences between the products, the device, and contextual factors. Furthermore, we checked whether pragmatic and hedonic quality explain variance in attractiveness.

Before analyzing the data, we performed several manipulation success checks (see Figure 6).

The first set of manipulation checks sought to identify which contextual factors significantly impacted UX. Given the high ecological validity we sought to achieve, these factors could relate to the tool, namely version (Version 1 vs. Version 2) and device (computer vs. tablet), as well as to other contextual factors, namely subsample (university vs. school) and setting (classroom or remote instruction during Covid-19 lockdowns). Thus, we first inspected boxplots of the UEQ results to identify outliers that might have an overly strong influence on the mean scores. Three potential outliers scored very low on the UEQ. However, when inspecting their concept maps, feedback, and scores on the other variables, the UEQ perfectly reflected their experience, as the three participants had severe problems with the tools. We concluded that these outliers do represent valid experience data, and thus decided to leave them in our data set. Second, we used histograms and Shapiro-Wilk tests (Ghasemi and Zahediasl, 2012; Shapiro and Wilk, 1965) to check the normal distribution assumption for the UEQ dimensions attractiveness, pragmatic, and hedonic quality. Attractiveness deviated from normal on computers, *W*(28) = 0.900, *p* = 0.011, and pragmatic quality deviated from normal in several settings, namely on tablets, *W*(43) = 0.941, *p* = 0.028, among school students, *W*(55) = 0.948, *p* = 0.019, and in in-person teaching settings, *W*(49) = 0.936, *p* = 0.011. Thus, and thirdly, we performed four independent t-tests using bootstrapping, assuming unequal variances (Field, 2018; Zimmerman, 2004) (see Table 5). Norman (2010) has pointed out that t-tests are generally considered robust, but in this paper, we used bootstrapping to overcome potential problems in the data (Field, 2018). Bootstrapping is a robust method to address problems of non-normality by repeatedly drawing random samples from the data (in our case, 1,000 times) to compute the relevant estimates of the tests. We used these estimates to calculate bias-corrected and accelerated confidence intervals rather than relying on a single test with the original sample (Efron and Tibshirani, 1993; Field, 2018). As an estimate of effect size, we calculated Cohen’s *d* (Cohen, 1988, 1992) using pooled standard deviations.

Besides the bootstrapping, we also performed a non-parametric Mann-Whitney U test for all tests where the UEQ dimensions were not normally distributed. Besides reporting the test outcome, we also calculated an effect size *r* as suggested by Field (2018). As Table 4 demonstrates, the results are identical to the t-tests with bootstrapping.

**Table 4:** Results of Mann-Whitney U tests.

Tested null hypothesis	Result of test	Conclusion
pragmatic quality is identical on each device (computer vs. tablet)	*U* = 371.5, *z* = -2.718, *p* = .007, *r* = -0.3226	reject the null hypothesis
attractiveness is identical on each device (computer vs. tablet)	*U* = 533.5, *z* = -.807, *p* = .420, *r* = -0.0958	retain the null hypothesis
pragmatic quality is identical for each population (school vs. university students)	*U* = 364.0, *z* = -1.046, *p* = .295, *r* = -0.1241	retain the null hypothesis
pragmatic quality is identical for each setting (remote vs. live)	*U* = 689.0, *z* = -1.872, *p* = .061, *r* = -0.2222	retain the null hypothesis

**Table 5:** t-tests of different factors on UEQ subscales.

Group	Pragmatic dimension	Hedonic dimension	Attractiveness
tool version (Tool 1 vs. Tool 2)	*t*(65.669) = 0.110 *p* = 0.913 *d* = 0.026	*t*(67.414) = 0.208 *p* = 0.836 *d* = 0.050	*t*(64.470) = -0.064 *p* = 0.949 *d* = -0.015
device (computer vs. tablet)	***t*(62.918) = 2.971** ***p* = 0.004** *d* = 0.702	*t*(52.244) = -0.622 *p* = 0.537 *d* = -0.155	*t*(61.619) = 1.162 *p* = 0.250 *d* = 0.277
population (university vs. school)	*t*(21.453) = 0.804 *p* = 0.430 *d* = 0.252	*t*(20.964) = 0.283 *p* = 0.780 *d* = 0.091	*t*(22.547) = 0.618 *p* = 0.543 *d* = 0.186
setting (remote vs. in-class)	*t*(52.540) = -1.732 *p* = 0.089 *d* = -0.401	*t*(45.138) = -0.002 *p* = 0.999 *d* = 0.000	*t*(45.368) = -0.556 *p* = 0.581 *d* = -0.136

The t-tests revealed that, contrary to our expectations, the different tool versions did not evoke significantly different user experiences. Interestingly, despite the lack of significant differences in user experience between the tool versions, the participants’ feedback on Tool 1 reflected aspects that were addressed in Tool 2 (see below for a detailed discussion). However, the device used for the concept mapping task had a significant impact. The pragmatic dimension of user experience was significantly higher on computers (*M* = 1.26, *SD* = 0.92) than on tablets (*M* = 0.56, *SD* = 1.05). The difference (0.70, +/- 0.45) was significant, *t*(62.918) = 2.97, *p* = 0.004, and represented a medium-sized effect of *d* = 0.70 (Cohen, 1988). The differences on the scales for attractiveness (computers: *M* = 0.92, *SD* = 1.16; tablets: *M* = 0.58, *SD* = 1.27) and hedonic dimension (computers: *M* = 0.24, *SD* = 1.32; tablets: *M* = 0.43, *SD* = 1.15) were not significant (see Table 5). No other differences were found.

In addition to the quantitative UX measurements obtained via the UEQ, we asked participants to provide feedback about the UX in freeform texts. This freeform text feedback provides insights into the reasons behind the participants’ reported user experience. Positive comments focused mostly on ease of use (3 mentions) or a generally pleasurable experience (10 mentions). Negative comments often focused on specific functionalities that participants experienced as annoying. On tablets, for example, the dedicated modes for selecting objects vs. zooming or scrolling were perceived as confusing (e.g., 14KM: *"when the scroll button is active I couldn't select any item. […] It is not practical to press a button each time I want to scroll”*). Thus, numerous suggestions referred to better scaling and scrolling options, in particular using finger gestures (e.g., 5LY, 14KM, 14TQ) or automatically selecting an object when it is tapped (regardless of which mode is active). These results indicate that the concept mapping prototypes might need to be more thoroughly adapted to multi-touch devices. The current solution with menus and dedicated modes might be acceptable on computers, but not on tablets, where more direct and enjoyable interactions might be more appropriate (Hwang et al., 2012). Thus, tablet interfaces are not more user-friendly per se, but must be specifically adapted to the interaction style inherent to this device.

Interestingly, although the differences on the UEQ between prototypes were insignificant, the participants’ feedback on the earlier tool nevertheless frequently reflected aspects that were addressed in the later tool, in particular providing onboarding instructions (e.g. 1SR: *“the presentation of the tool was good”*), making it possible to customize colors and fonts, or removing the ability to switch between modes for creating single or unlimited objects. One of the most frequent suggestions on tablets was an alternative interaction style involving drawing a concept map with a stylus (e.g. 25LD: *“draw more with a pencil on the tablet, and then the application would render the shapes more attractive so that I would not have to select different tools without end, as this is annoying and takes time”*). This feedback suggests that the current free-hand drawing tool focused on adding simple annotations is too limited.

**Figure 7:**
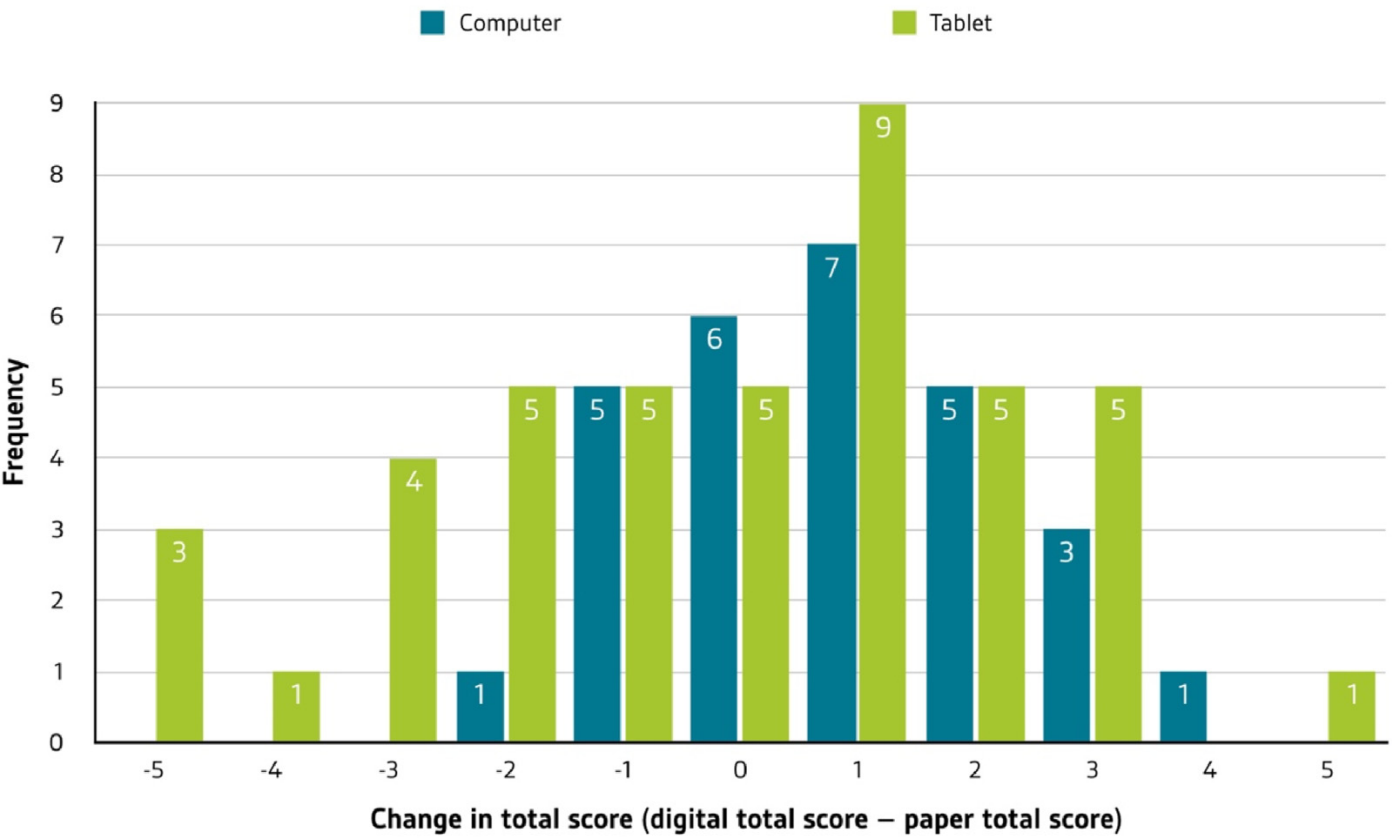
Changes in total scores on computers and tablets. Diagram of the changes in score on computers and tablets (as compared to the paper version). Most students were able to achieve slightly higher scores.

Table 6 provides descriptive statistics for the measured variables. Needs fulfillment and intention to use were relatively high, while concept map scores showed a normal distribution. Overall, the mean scores on the digital concept maps resembled those on the paper concept maps. However, when exploring the individual change scores (per participant), it became apparent that the majority of learners achieved higher scores on the digital concept maps, particularly on computers (see Figure 7).

**Table 6:** Descriptive statistics for the variables in the present study.

Variable	Computer (*N* = 28)	Tablet (*N* = 43)
*Needs fulfillment (0–5 scale)*		
Autonomy & independence	*M* = 3.67, *SD* = 0.75	*M* = 3.22, *SD* = 0.84
Competence & effectiveness	*M* = 3.34, *SD* = 0.93	*M* = 3.11, *SD* = 0.92
Relatedness & belongingness	*M* = 2.83, *SD* = 0.94	*M* = 2.48, *SD* = 1.02
Pleasure & stimulation	*M* = 3.13, *SD* = 0.83	*M* = 2.79, *SD* = 0.96
Security & control	*M* = 3.46, *SD* = 0.74	*M* = 3.17, *SD* = 0.85
Influence & popularity	*M* = 2.96, *SD* = 0.76	*M* = 2.70, *SD* = 0.73
Self-realization & meaning	*M* = 3.48, *SD* = 1.00	*M* = 2.93, *SD* = 0.86
Global needs	*M* = 3.27, *SD* = 0.65	*M* = 2.93, *SD* = 0.73
*Intention to use (0–5 scale)*	*M* = 3.17, *SD* = 1.30	*M* = 2.93, *SD* = 1.19
*Scores*		
Comprehensiveness (0–3 scale)	*M* = 1.57, *SD* = 0.60 (Paper map: *M* = 1.42, *SD* = 0.47)	*M* = 1.70, *SD* = 0.64 (Paper map: *M* = 1.77, *SD* = 0.55)
Organization (0–3 scale)	*M* = 1.50, *SD* = 0.61 (Paper map: *M* = 1.25, *SD* = 0.40)	*M* = 1.51, *SD* = 0.81 (Paper map: *M* = 1.55, *SD* = 0.50)
Correctness (0–3 scale)	*M* = 1.66, *SD* = 0.61 (Paper map: *M* = 1.34, *SD* = 0.45)	*M* = 1.50, *SD* = 0.72 (Paper map: *M* = 1.56, *SD* = 0.47)
Holistic total score (0–9 scale)	*M* = 4.73, *SD* = 1.60 (Paper map: *M* = 4.02, *SD* = 1.04)	*M* = 4.47, *SD* = 1.94 (Paper map: *M* = 4.86, *SD* = 1.15)

Finally, to check whether hedonic and pragmatic UX predict attractiveness, we calculated two linear regression models. The results indicated that pragmatic and hedonic qualities of user experience significantly predicted attractiveness in both conditions (computers: *F*(2, 25) = 73.874, *p* = 0.000, tablets: *F*(2,40) = 80.570, *p* = 0.000). The overall fit of the models was very good (computers: *R*^*2*^ = 0.86, tablets: *R*^*2*^ = 0.80). The model parameters on computers (all *p* = 0.000) were *b* = 0.55 for pragmatic quality (SE 0.11; +/- 0.23) and *b* = 0.57 for hedonic quality (SE 0.07; +/- 0.15). The model parameters on tablets (all *p* = 0.000) were *b* = 0.45 for pragmatic quality (SE 0.11; +/- 0.11) and *b* = 0.668 for hedonic quality (SE 0.10; +/- 0.35). Therefore, we concluded that the UEQ reports UX as expected.

### 3.6 Checks of bias and assumptions in linear regression models

We investigated our research questions with the help of linear regression models: For RQ 1, we used linear regression models with need fulfillment regressed on both pragmatic and hedonic UX. For RQs 2 and 3, we used linear regression models with attractiveness regressed on intention to use and the four assessment scores. We checked each of these models for bias and assumptions, as suggested by Field (2018). These checks serve to assess whether the models generalize. We want to first report on these checks before reporting the results.

First, we checked case-wise diagnostics for standardized residuals. Field (2018) suggests that 95% of cases should be within two standard deviations in a normally distributed sample. In our sample, we expect this to be true for 26–27 cases in the computer group (N = 28) and 40–41 cases in the tablet group (N = 43). Table 7 shows how many cases in each model fell within this expected range. The model predicting pragmatic UX from the fulfillment of pleasure and stimulation on tablets is the only model outside the expected range, with four cases more than -2 standard deviations from the expected value. However, two of these cases were close to -2 standard deviations, with z-scores of -2.026 and -2.043, respectively. Thus, we do not consider these values to indicate major bias in the model.

**Table 7:** Casewise diagnostics.

Linear model	Cases inside the expected range
Global need → pragmatic UX Global need → hedonic UX	Computers: 28, Tablets: 41 Computers: 27, Tablets: 42
Autonomy → pragmatic UX Autonomy → hedonic UX	Computers: 27, Tablets: 40 Computers: 27, Tablets: 41
Competence → pragmatic UX Competence → hedonic UX	Computers: 27, Tablets: 42 Computers: 27, Tablets: 42
Relatedness → pragmatic UX Relatedness → hedonic UX	Computers: 28, Tablets: 41 Computers: 28, Tablets: 42
Pleasure → pragmatic UX Pleasure → hedonic UX	Computers: 27, Tablets: 39 Computers: 27, Tablets: 42
Security → pragmatic UX Security → hedonic UX	Computers: 27, Tablets: 42 Computers: 28, Tablets: 42
Influence → pragmatic UX Influence → hedonic UX	Computers: 28, Tablets: 41 Computers: 27, Tablets: 41
Self-actualizing → pragmatic UX Self-actualizing → hedonic UX	Computers: 27, Tablets: 40 Computers: 27, Tablets: 42
Attractiveness → intention to use	Computers: 27, Tablets: 40
Attractiveness → comprehensiveness score	Computers: 26, Tablets: 42
Attractiveness → organization score	Computers: 27, Tablets: 41
Attractiveness → correctness score	Computers: 27, Tablets: 41
Attractiveness → total score	Computers: 26, Tablets: 41

**Figure 8:**
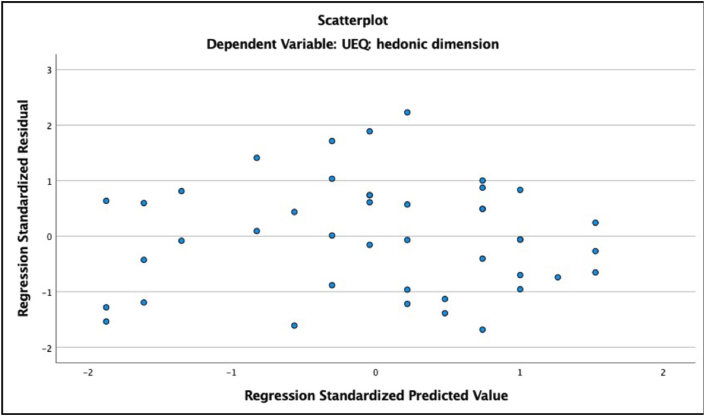
ZResid vs. ZPred for the model with fulfillment of need for pleasure and stimulation predicting the hedonic quality of UX. Example of random distribution of ZResid and ZPred scores.

**Figure 9:**
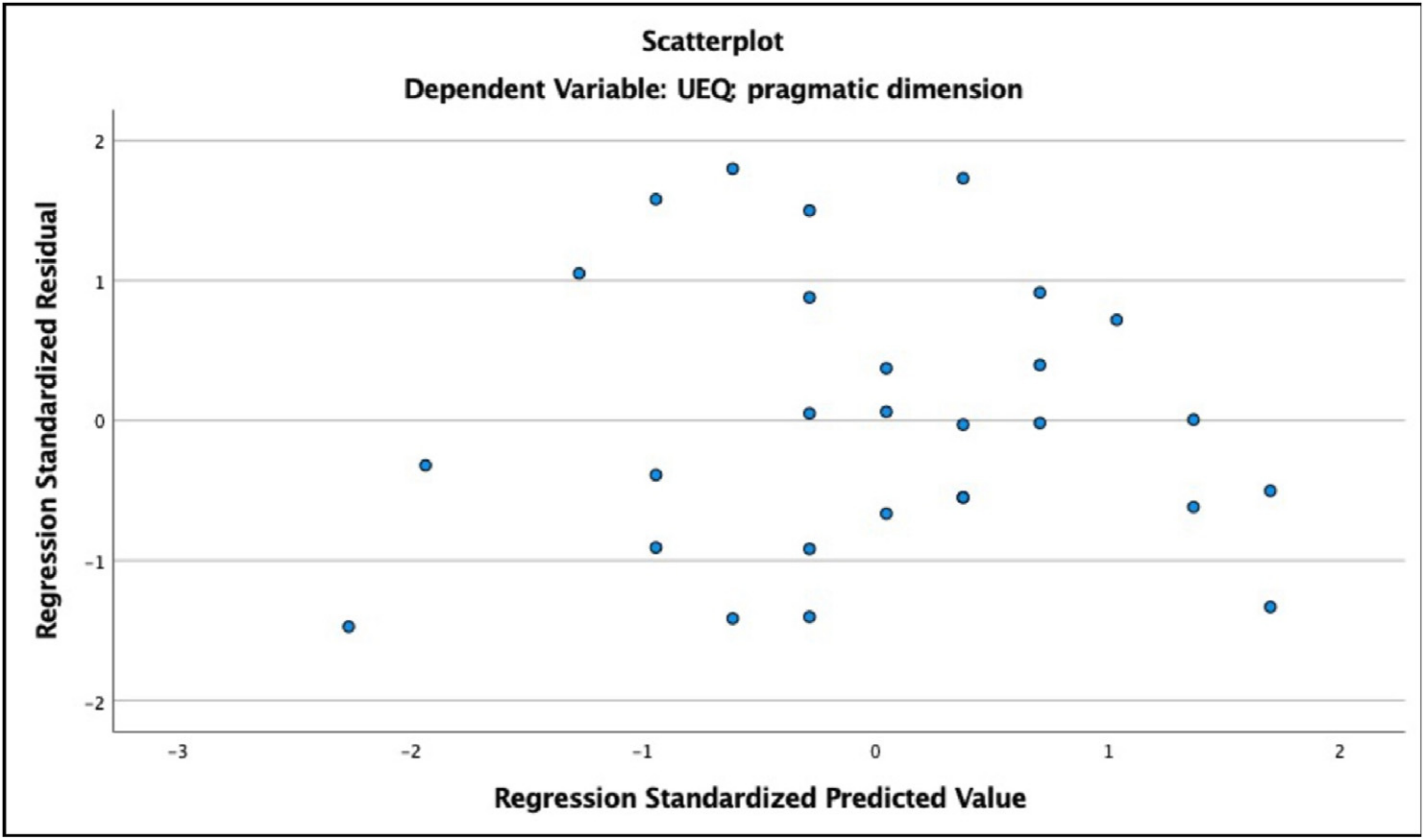
ZResid vs. ZPred for the model with fulfillment of the need for influence and popularity predicting the pragmatic quality of UX. Funnel-like distribution of ZResid and ZPred scores.

**Figure 10:**
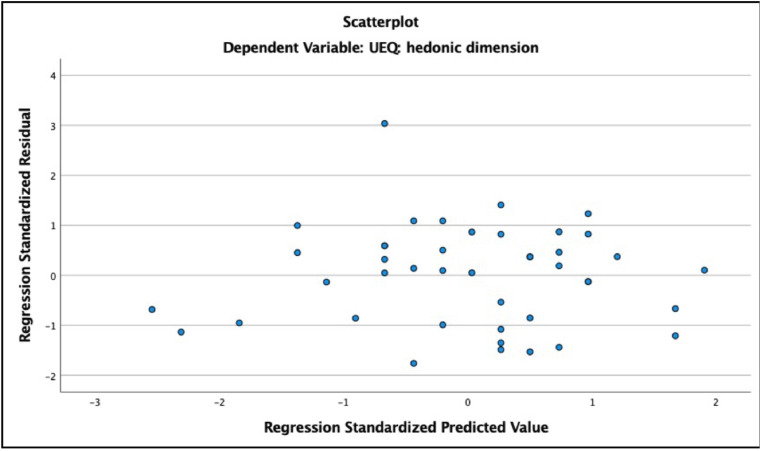
ZResid vs. ZPred of the model with fulfillment of the need for security and control predicting the hedonic quality of UX. Funnel-like distribution of ZResid and ZPred scores.

Second, we checked for signs of heteroscedasticity and non-normal distribution of residuals (Field, 2018). For heteroscedasticity, we checked scatterplots of standardized predicted values against standardized residuals for each of our models (ZResid vs. ZPred). Heteroscedasticity would be reflected in graphs with a funnel-like pattern in which values become more spread out. Most of our models exhibited a random distribution of values, indicating that the homoscedasticity assumption is met (see Figure 8 for an example). The graph for the model with the need fulfillment of influence and popularity predicting the pragmatic dimension of UX shows a clear funnel-like pattern indicative of heteroscedasticity (see Figure 9). However, we suggest treating the results for social needs with caution in the present study (see discussion section) as the task did not involve social interaction. The graph for the model with the need fulfillment of security and control predicting the hedonic dimension of UX exhibited a similar pattern (see Figure 10), suggesting that this model should also be interpreted with caution.

To test for non-normal distributions of residuals, we checked histograms and normal probability plots (P–P plots; Field, 2018). A minority of histograms and P–P plots suggested some concerns regarding the normality of residuals, particularly in the computer group (models: attractiveness → organization score, pragmatic dimension of UX → security). Consequently, we again performed bootstrapping to obtain robust confidence intervals and significance tests (Field, 2018).

Finally, we cross-validated the significant models to verify generalizability. Specifically, we used Stein’s formula as suggested by Field (2018), where *n* refers to the sample size (28 for the computer and 43 for the tablet groups) and *k* refers to the number of predictors (1 for our models):

$$adjusted\;R^{2}=1-\;\left[\left(\frac{n-1}{n-k-1}\right)\left(\frac{n-2}{n-k-2}\right)\left(\frac{n+1}{n}\right)\right]\left(1-R^{2}\right)\;$$

These adjusted R^2^ values indicate how much predictive power is lost “if the model had been derived from the population from which the sample was taken” (Field, 2018, p. 389). We will discuss these adjusted R^2^ values and other considerations about generalizing our findings in the discussion section.

## 4 Results and discussion

### 4.1 Research question 1: impact of needs fulfillment on pragmatic and hedonic UX

To examine the influence of need fulfillment on the pragmatic and hedonic subdimensions of UX (RQ 1), we calculated linear regression models with the fulfillment of all seven measured needs regressed on pragmatic (see Table 8) and hedonic UX (see Table 9). The results indicated that autonomy and independence had the strongest explanatory power for positive UX in digital concept mapping, with respect to both the pragmatic (computers: 54.4%, tablets: 26.0%) and the hedonic dimension (computers: 23.8%, tablets: 32.8%). A good digital concept mapping tool allows learners to create concept maps without external help or training (“do-goal”) and provides them with the functionalities they need to express themselves as desired (“be-goal”; Hassenzahl, 2008). These autonomy-supporting characteristics result in a positive UX. This interpretation is supported by the high explanatory power of fulfillment of need for security/control for pragmatic UX (computers: 52.6%, tablets: 37.8%), indicating that when learners feel in control of the interaction, they have a positive experience regarding the achievement of their “do-goals”. It has been suggested that security and control should be considered a “deficiency need, i.e., a need that creates negative affect if blocked, but not necessarily strong positive feelings if fulfilled” (Hassenzahl et al., 2010, p. 358). Thus, we would expect security and control to explain variance in pragmatic UX but not hedonic UX. However, security/control also explained 37.8% of the variance in hedonic UX on tablets, although it was insignificant on computers. Potentially, the lower overall pragmatic quality of the tablet optimization led to lower fulfillment of “be-goals”, but the better overall pragmatic quality of our computer prototypes had no positive impact on the experience of achieving “be-goals”. However, we also note that the model with security/control predicting hedonic UX should be interpreted with caution because the assumption of homoscedasticity (Field, 2018) might not hold (see Section 3.6).

**Table 8:** Explanatory power of need fulfillment on pragmatic UX.

Predictor of pragmatic UX	R^2^	*b*	SE *b*	*p*
autonomy and independence	**Computers: 0.544** **Tablets: 0.260**	**Computers: 0.904 (+/- 0.482)** **Tablets: 0.633 (+/- 0.307)**	**Computers: 0.176** **Tablets: 0.184**	**Computers: 0.000** **Tablets: 0.000**
competence and effectiveness	**Computers: 0.363** **Tablets: 0.167**	**Computers: 0.599 (+/- 0.269)** **Tablets: 0.466 (+/- 0.382)**	**Computers: 0.162** **Tablets: 0.219**	**Computers: 0.001** **Tablets: 0.006**
relatedness and belongingness	Computers: 0.072 Tablets: 0.056	Computers: 0.263 (+/- 0.366) Tablets: 0.243 (+/- 0.304)	Computers: 0.204 Tablets: 0.148	Computers: 0.168 Tablets: 0.126
pleasure and stimulation	Computers: 0.085 **Tablets: 0.190**	Computers: 0.322 (+/- 0.541) **Tablets: 0.478 (+/- 0.316)**	Computers: 0.226 **Tablets: 0.166**	Computers: 0.132 **Tablets: 0.003**
security and control	**Computers: 0.526** **Tablets: 0.378**	**Computers: 0.903 (+/- 0.331)** **Tablets: 0.754 (+/- 0.247)**	**Computers: 0.175** **Tablets: 0.167**	**Computers: 0.000** **Tablets: 0.000**
influence and popularity	**Computers: 0.267** Tablets: 0.042	**Computers: 0.629 (+/- 0.385)** Tablets: 0.291 (+/- 0.512)	**Computers: 0.211** Tablets: 0.214	**Computers: 0.005** Tablets: 0.190
self-realization and meaning	**Computers: 0.155** **Tablets: 0.129**	**Computers: 0.361 (+/- 0.616)** **Tablets: 0.437 (+/- 0.369)**	**Computers: 0.267** **Tablets: 0.214**	**Computers: 0.038** **Tablets: 0.018**

**Table 9:** Explanatory power of need fulfillment on hedonic UX.

Predictor of hedonic UX	R^2^	b	SE b	p
autonomy and independence	**Computers: 0.238** **Tablets: 0.328**	**Computers: 0.856 (+/- 0.53)** **Tablets: 0.783 (+/- 0.249)**	**Computers: 0.285** **Tablets: 0.158**	**Computers: 0.008** **Tablets: 0.000**
competence and effectiveness	Computers: 0.069 **Tablets: 0.180**	Computers: 0.373 (+/- 0.57) **Tablets: 0.532 (+/- 0.338)**	Computers: 0.304 **Tablets: 0.209**	Computers: 0.177 **Tablets: 0.005**
relatedness and belongingness	Computers: 0.009 **Tablets: 0.119**	Computers: 0.130 (+/- 0.463) **Tablets: 0.390 (+/- 0.311)**	Computers: 0.263 **Tablets: 0.165**	Computers: 0.640 **Tablets: 0.023**
pleasure and stimulation	**Computers: 0.188** **Tablets: 0.297**	**Computers: 0.684 (+/- 0.599)** **Tablets: 0.657 (+/- 0.247)**	**Computers: 0.290** **Tablets: 0.145**	**Computers: 0.021** **Tablets: 0.000**
security and control	Computers: 0.062 **Tablets: 0.378**	Computers: 0.445 (+/- 0.735) **Tablets: 0.829 (+/- 0.234)**	Computers: 0.317 **Tablets: 0.152**	Computers: 0.200 **Tablets: 0.000**
influence and popularity	Computers: 0.020 **Tablets: 0.124**	Computers: 0.247 (+/- 0.661) **Tablets: 0.553 (+/- 0.46)**	Computers: 0.328 **Tablets: 0.236**	Computers: 0.472 **Tablets: 0.021**
self-realization and meaning	Computers: 0.124 **Tablets: 0.226**	Computers: 0.455 (+/- 0.523) **Tablets: 0.636 (+/- 0.353)**	Computers: 0.216 **Tablets: 0.186**	Computers: 0.066 **Tablets: 0.001**

Certain needs systematically explained variance in pragmatic UX, namely competence/effectiveness (computers: 36.3%, tablets: 16.7%) and self-actualizing/meaning (computers: 15.5%, tablets: 12.9%). Both needs also explained variance in hedonic UX, but only on tablets (18.0% and 22.6%, respectively). When a digital concept mapping tool allows learners to successfully express their cognitive structures (Ifenthaler, 2010), they feel competent and realize their full potential, resulting in a positive UX. Mekler and Hornbæk (2016) reported correlations between pragmatic and eudaimonic qualities which might explain why the learning-related needs were particularly predictive of pragmatic UX.

Turning to hedonic UX, the need for pleasure and stimulation significantly explained variance in hedonic UX (computers: 18.8%, tablets: 29.7%) and thus seems to be related to participants’ “be-goals” (Hassenzahl, 2008). Interestingly, pleasure had greater explanatory power on tablets than on computers. Pleasure and stimulation also predicted variance in pragmatic UX (19.0%), but only on tablets. We hypothesize that this explanatory power on tablets might be related to the different style of interaction, which has been described as more enjoyable (Hwang et al., 2012). Thus, designing a tablet tool with a positive UX has the potential to better fulfil learners’ needs for pleasure and stimulation. Computers, on the other hand, could be a more “neutral” device with a stronger focus on pragmatic qualities. Thus, impressions of need fulfillment might vary between computers and tablets, further indicating the need to optimize user experience for each device separately.

In summary, we found evidence supporting Hypothesis 1: Our results suggest that need fulfillment significantly explains variance in pragmatic and hedonic UX, although not universally for all needs and devices. This finding is further supported by the importance ratings participants gave each need for digital concept mapping (see Table 10). The most important needs for digital concept mapping are security/control, competence/effectiveness, pleasure/stimulation, autonomy/independence, and self-realization/meaning. These results can serve as a starting point for “compil[ing] a product-specific needs profile” (Desmet and Fokkinga, 2020, p. 11) for concept mapping. The social needs of relatedness/belongingness and influence/popularity were given lower importance ratings and played a smaller role in explaining variance in UX, likely due to the individual nature of the concept mapping setting in the present study. The results might be different for collaborative concept mapping activities (Khamesan and Hammond, 2004). Independent samples t-tests revealed no significant differences in the importance of psychological needs between devices.

**Table 10:** Descriptive statistics on the importance of needs.

Need	Computer	Tablet
Autonomy/independence	*M* = 3.21, *SD* = 0.74	*M* = 2.95, *SD* = 0.94
Competence/effectiveness	*M* = 3.37, *SD* = 1.08	*M* = 3.30, *SD* = 0.71
Relatedness/belongingness	*M* = 2.25, *SD* = 1.08	*M* = 2.60, *SD* = 0.82
Pleasure/stimulation	*M* = 3.25, *SD* = 0.89	*M* = 3.14, *SD* = 0.97
Security/control	*M* = 3.46, *SD* = 0.64	*M* = 3.33, *SD* = 0.75
Influence/popularity	*M* = 2.46, *SD* = 1.10	*M* = 2.70, *SD* = 0.91
Self-realization/meaning	*M* = 3.11, *SD* = 0.88	*M* = 3.14, *SD* = 0.80

### 4.2 Research questions 2 and 3: impact of user experience on intention to use and digital concept mapping scores

To determine the impact of user experience on intention to use (RQ 2) and assessment scores (RQ 3), we calculated linear regression models with attractiveness predicting intention to use and the four assessment scores. The results for intention to use (see Table 11) confirm that user experience is vital for acceptance of a digital product. The UX dimension of attractiveness significantly predicted 72.3% of intention to use on computers and 36.5% of intention to use on tablets. Thus, we found no evidence that would lead us to reject Hypothesis 2 and concluded that UX significantly impacts intention to use. Interestingly, the amount of variance explained by attractiveness is much higher on computers than on tablets. Potentially, the pragmatic issues on tablets impacted intention to use. Alternatively, there might be general differences in technology acceptance, with a specific group of users rejecting tablets for digital concept mapping in general (Amadieu et al., 2019).

**Table 11:** Models using attractiveness as a predictor for the respective outcome variables.

Outcome	R2	b	SE b	p
Intention to use	**Computers: 0.723** **Tablets: 0.365**	**Computers: 0.906 (+/- 0.217)** **Tablets: 0.544 (+/- 0.218)**	**Computers: 0.111** **Tablets: 0.101**	**Computers: 0.000** **Tablets: 0.000**
Change in comprehensiveness scores	Computers: 0.011 Tablets: 0.063	Computers: -0.042 (+/- 0.150) Tablets: 0.163 (+/- 0.219)	Computers: 0.064 Tablets: 0.105	Computers: 0.600 Tablets: 0.104
Change in organization scores	Computers: 0.053 **Tablets: 0.091**	Computers: -0.116 (+/- 0.173) **Tablets: 0.198 (+/- 0.175)**	Computers: 0.084 **Tablets: 0.085**	Computers: 0.240 **Tablets: 0.049**
Change in correctness scores	Computers: 0.041 **Tablets: 0.109**	Computers: 0.109 (+/- 0.207) **Tablets: 0.233 (+/- 0.212)**	Computers: 0.101 **Tablets: 0.113**	Computers: 0.303 **Tablets: 0.031**
Change in total score	Computers: 0.002 **Tablets: 0.112**	Computers: -0.049 (+/- 0.287) **Tablets: 0.594 (+/- 0.563)**	Computers: 0.179 **Tablets: 0.278**	Computers: 0.828 **Tablets: 0.028**

Turning to the influence of attractiveness on concept map scores (Hypothesis 3), we found that attractiveness explained variance in changes in organization scores, changes in correctness scores, and changes in total scores, with small *R*^*2*^ values ranging from 0.091 to 0.112 on tablets (see Table 11). The models for changes in comprehensiveness scores on tablets and the models for all change scores on computers were not significant, indicating the weak explanatory power of attractiveness. Thus, we concluded that the evidence for rejecting Hypothesis 3 is inconclusive: attractiveness significantly explains variance in three of the four scores on tablets, but does not explain variance in comprehensiveness scores or on computers.

One explanation for the low explanatory power of UX for concept map scores could be that the different qualities of UX do not impact concept map scores evenly. When pragmatic quality is low, learners might need to invest cognitive resources in using the tool instead of the task, in line with cognitive load theory (Sweller, 1994; Sweller et al., 2011). Interestingly, our results suggest that a threshold of pragmatic quality might exist, with pragmatic quality only impacting scores when it is below the hypothesized threshold. In line with this interpretation, UX had no explanatory power on computer versions of our tools, where the pragmatic quality was higher. Additionally measuring cognitive load might help to assess whether this interpretation holds (Amadieu et al., 2009).

Regarding hedonic quality, we think that it is too early to draw a conclusion. The theorized positive impact of hedonic quality might potentially develop over time, as higher engagement leads to a gradual improvement in scores. Furthermore, the hedonic qualities of our tools did not differ significantly and were largely in a medium range (Schrepp et al., 2017). Potentially, studies of repeated tool use or tools with higher overall hedonic qualities might be needed to uncover the impact of hedonic qualities on concept map scores. It is also possible that hedonic quality is related to learning-related outcomes other than assessment scores, such as completion rates.

### 4.3 Conclusion

As learning and assessment are becoming increasingly digitalized, it is vital to explore learners’ experiences with these digital tools. The present paper, based on a field study in three schools and a university (N = 71), found that user experience (UX) significantly explained variance in intention to use a digital concept mapping tool. UX was also capable of explaining variance in some concept mapping scores. Furthermore, fulfillment of psychological needs was found to be an important driver of users’ experience with this digital technology. Thus, UX is important for providing a positive and successful environment for digital concept mapping.

Our results have a range of implications beyond digital concept mapping. With respect to the design and evaluation of digital education products, our findings suggest that tools should be optimized for each particular technological context in order to provide an adequate user experience, just as educational tasks are adapted to the devices used (Mulet et al., 2019). Good solutions on computers do not necessarily work equally well on tablets. These results further suggest that user experience investigated in one technological context cannot necessarily be transferred to another.

Our results support UX as a key concept for digital education products and indicate that UX models can be used to predict outcome variables similarly to technology acceptance models (Šumak et al., 2011). UX could advance the discussion on technology acceptance because it is rooted in concrete experiences and design solutions (Amadieu et al., 2019).

Our study provided some evidence that UX can explain variance in concept map scores, but the finding did not hold for all conditions. Given the growing importance of assessment with digital technologies (Redecker and Johannessen, 2013; Ng, 2015), research should systematically investigate the impact of UX on such assessment scores to ensure fair conditions for learners and create positive experiences of learning with technology.

#### 4.3.1 Generalizability of our findings

An important consideration is whether our results generalize to a population beyond our sample. In summary, we think that our sample is indicative of our target audience, although we suggest being cautious when generalizing to an audience with different characteristics. In the following, we discuss the generalizability of our findings regarding three considerations.

First, our study is a field study with a sample size of 71. The determination of sample size was mainly driven by a feasibility analysis (Caine, 2016). As briefly outlined in Section 3, this study is part of a research project on UX in digital concept mapping. Prior to this study, we conducted several rounds of lab-based user testing with earlier prototypes, but we wanted to extend our findings to a field setting to raise ecological validity. However, our prototypes are not mature products that we could roll out to schools without close attendance from our side. Thus, a field study allowed us to investigate user experience in a realistic, standardized setting while collecting participants’ qualitative feedback and providing a detailed debriefing. However, this decision required detailed preparation, such as acquiring approval and informing instructors, parents, and students ahead of the study, and thus constrained the feasibility of a large-scale, survey-based study. Such a study would be very interesting as a follow-up because we are currently rolling out a mature concept mapping tool based on our research findings in Luxembourg. However, the sample size of 71 is not unusually small for such a study. For example, in Human-Computer Interaction, Caine (2016) observed mean sample sizes for field studies of 19 for in-person and 89 for remote settings.

Second, we performed cross-validation (see Section 3.6) of the significant models using Stein’s adjusted R^2^ as suggested by Field (2018). Table 12 shows the results. We generally observe a low loss of predictive power in the adjusted R^2^, except for the models predicting scores from attractiveness. These findings are in line with the inconclusive evidence regarding our Hypothesis 3.

**Table 12:** Cross-validation of models.

Model	R^2^	adjusted R^2^
autonomy/independence → pragmatic UX	Computers: 0.544 Tablets: 0.260	Computers: 0.490 Tablets: 0.205
competence/effectiveness → pragmatic UX	Computers: 0.363 Tablets: 0.167	Computers: 0.287 Tablets: 0.105
pleasure/stimulation → pragmatic UX	Tablets: 0.190	Tablets: 0.130
security/control → pragmatic UX	Computers: 0.526 Tablets: 0.378	Computers: 0.470 Tablets: 0.332
influence/popularity → pragmatic UX	Computers: 0.267	Computers: 0.180
self-realization/meaning → pragmatic UX	Computers: 0.155 Tablets: 0.129	Computers: 0.055 Tablets: 0.064
autonomy/independence → hedonic UX	Computers: 0.238 Tablets: 0.328	Computers: 0.148 Tablets: 0.278
competence/effectiveness → hedonic UX	Tablets: 0.180	Tablets: 0.112
relatedness/belongingness → hedonic UX	Tablets: 0.119	Tablets: 0,053
pleasure/stimulation → hedonic UX	Computers: 0.188 Tablets: 0.297	Computers: 0.091 Tablets: 0.245
security/control → hedonic UX	Tablets: 0.378	Tablets: 0.332
influence/popularity → hedonic UX	Tablets: 0.124	Tablets: 0.058
self-realization/meaning → hedonic UX	Tablets: 0.226	Tablets: 0.168
attractiveness → intention to use	Computers: 0.723 Tablets: 0.365	Computers: 0.690 Tablets: 0.318
attractiveness → change in organization	Tablets: 0.091	Tablets: 0.023
attractiveness → change in correctness	Tablets: 0.109	Tablets: 0.043
attractiveness → change in total	Tablets: 0.112	Tablets: 0.046

Third, we want to discuss qualitative considerations regarding the generalizability. As indicated, we recruited our test participants based on previous studies in a larger research project investigating the user experience of digital concept mapping in Luxembourg. The main audience we identified in these studies are students from secondary and tertiary education who have little experience with digital concept mapping. Thus, we are confident that they are indicative of the target audience for our setting. However, the results might differ for other populations, such as people with more experience in concept mapping. It would be interesting to replicate our study in another setting. For example, experienced users might have less pragmatic issues because of being more used to a particular concept mapping tool. Consequently, need fulfillment might be more important for explaining variance in hedonic user experience for these people rather than in pragmatic user experience as in our sample.

#### 4.3.2 Design recommendations

Several recommendations for the design of digital assessment and learning tools can be derived from our study as well. First, we recommend that designers consider the role and importance of individual psychological needs with regard to the product or service they are designing. Such a “product-specific needs profile” (Desmet and Fokkinga, 2020, p. 11) could potentially serve as a useful guideline for designing a positive user experience (which in turn impacts intention to use). Second, we found evidence that experience is strongly impacted by technology. The found differences between tablets and computers suggest that it is necessary to account for technology-specific adaptations like touchscreen-based interaction patterns to provide equally positive experiences. Such adaptations appear to be worthwhile because, third, we found strong support for viewing UX as a success factor for digital products. Fourth, our results indicate that UX might have an impact on outcomes, namely the scores achieved in concept map-based assessment. Although our results suggest a need for further research into why and when UX impacts assessment outcomes, the growing digitalization of education makes it necessary to consider products from a design-driven perspective to ensure that they provide appropriate circumstances for learning and assessment.

#### 4.3.3 Limitations

The present study has four limitations. First, although most ω values were acceptable, the value for influence and popularity (ω = 0.61) was relatively low. However, this particular need was also rated as relatively unimportant for digital concept mapping and therefore only played a minor role in our setting.

Second, although the majority of learners were able to gain new knowledge, scores on the paper concept maps (pre-learning) and digital concept maps (post-learning) were relatively close to each other. Thus, the incentive to learn may have been low and might not generalize to situations in which the concept mapping scores affect students’ grades (Heidig et al., 2015). However, studying the impact of user experience on concept map scores in a higher-stakes situation for participants would pose ethical challenges: Design issues with the concept mapping tool might systematically penalize certain groups of learners. Thus, we consider the relatively low overall learning success acceptable for the purpose of this study.

Third, UX is primarily related to perceived qualities (Hassenzahl, 2010). However, less subjective factors like the time needed to create a proposition might also play a role, particularly when it comes to assessment scores. Therefore, it might be worthwhile to replicate this study with additional objective measurements of the interaction such as a log system. This approach would allow researchers to triangulate participants’ subjective evaluations with their objective behavior.

Fourth, we used a single item for measuring intention to use, based on reflections and pre-testing as outlined in Section 3.4. However, we did not systematically investigate whether multiple items would be preferable. Multiple items are used in research in the tradition of the Technology Acceptance Model (Venkatesh et al., 2003) or in the meCUE questionnaire (Minge et al., 2016). It would be interesting to investigate whether intention to use should best be measured with a single item or multiple items, in particular to verify whether intention to use is a “double concrete” construct (Rossiter, 2002) or whether it covers multiple facets that should be considered.

## Declarations

### Author contribution statement

Björn Rohles, Susanne Backes: Conceived and designed the experiments; Performed the experiments; Analyzed and interpreted the data; Contributed reagents, materials, analysis tools or data; Wrote the paper.

Antoine Fischbach, Vincent Koenig: Conceived and designed the experiments; Contributed reagents, materials, analysis tools or data; Wrote the paper.

Franck Amadieu: Conceived and designed the experiments; Wrote the paper.

### Funding statement

This research was funded by the SCRIPT, an institution of the Luxembourg government to enhance education.

### Data availability statement

Data will be made available on request.

### Declaration of interests statement

The authors declare no conflict of interest.

### Additional information

No additional information is available for this paper.

## Figures and Tables

**Figure:**
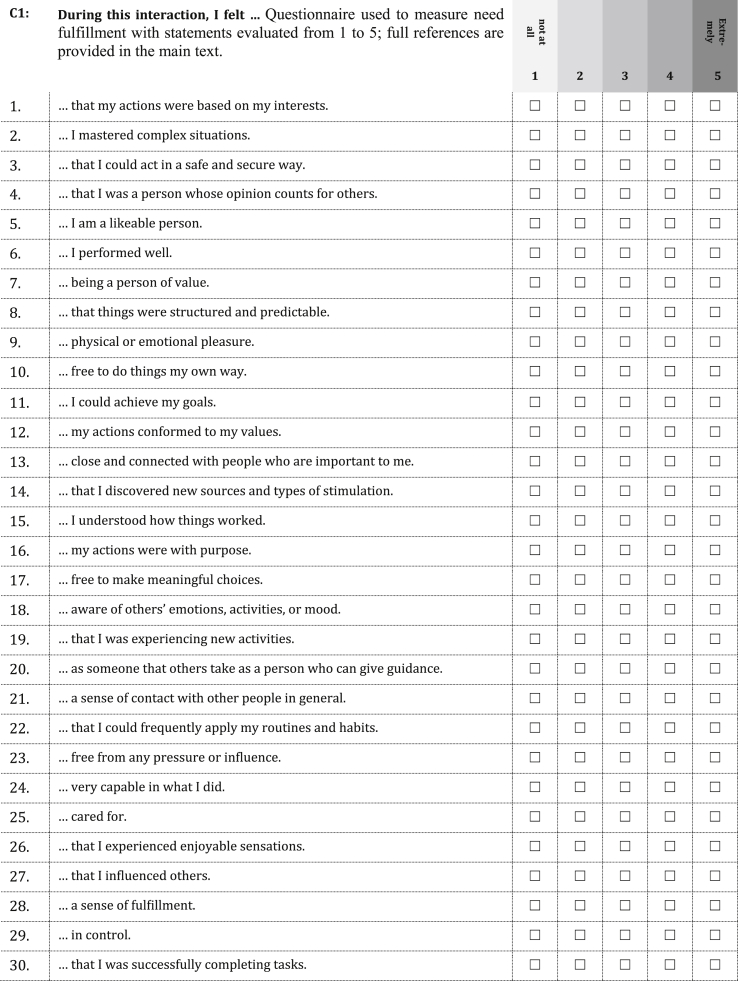


**Figure:**
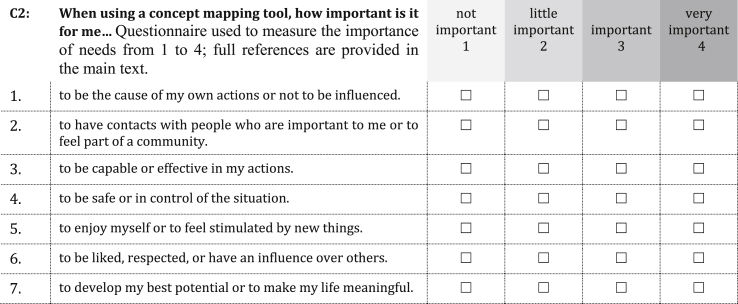


**Figure:**
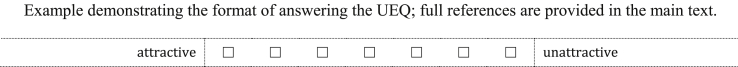


**Figure:**
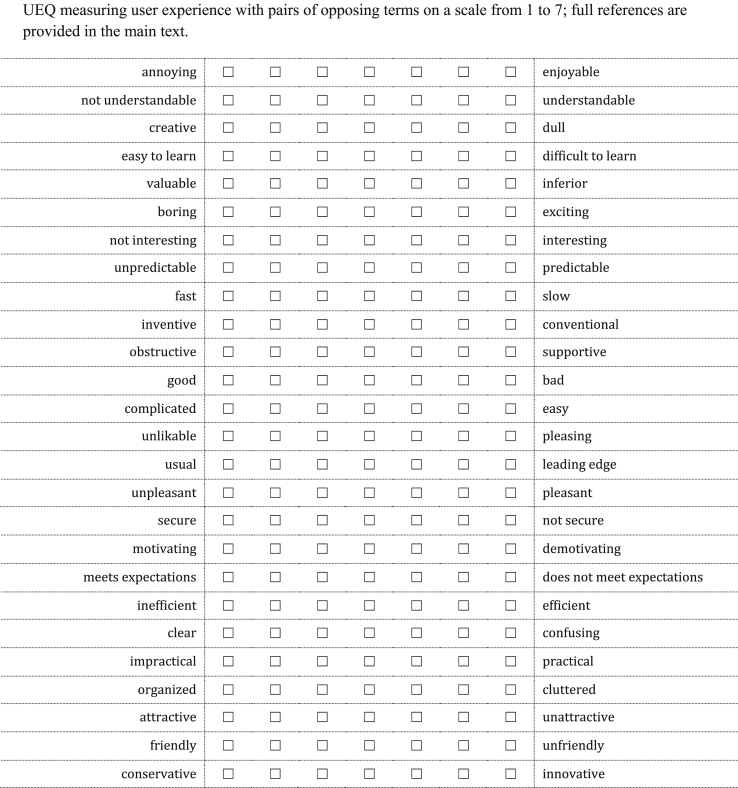


**Figure:**
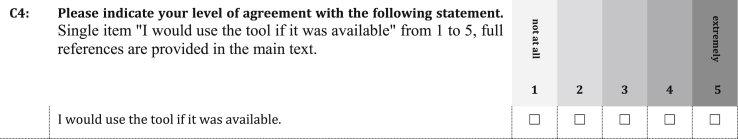


**Figure:**
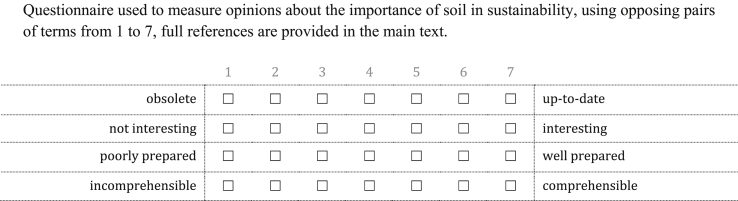


**Figure:**
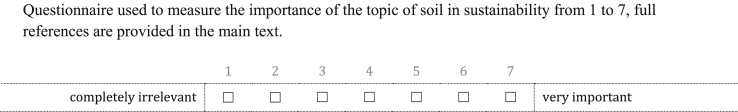


**Figure:**
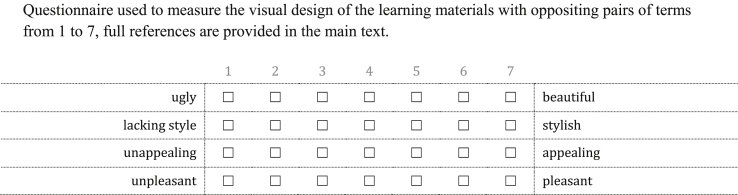


**Figure:**
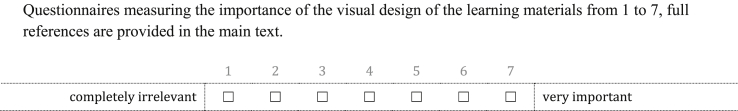


## Appendix

### Measurement instruments

On the following pages, we provide the measurement instruments used in our study. C1 is the measurement of need fulfillment, developed by Lallemand and Koenig (2017) and based on Sheldon et al. (2001). C2 assesses the importance of each need for digital concept mapping, again developed by Lallemand and Koenig (2017). C3 is the UEQ as a measurement of user experience of the concept mapping tools (Laugwitz et al., 2008). C4 is our measurement of intention to use, C5 is a free text field for qualitative feedback about the tools. Finally, C6 to C9 are derived from the scales of content quality and visual quality from UEQ+, a modular extension of UEQ (Schrepp and Thomaschewski, 2019). We used them to check the perceived quality of the learning materials.

C1: The following questionnaire contains descriptions of complex feelings that we ask you to rate relative to your interaction with the concept mapping tool. All the sentences start with “During this interaction, I felt…”. Please rate each sentence on a scale ranging from 1 Not at all to 5 Extremely.

C2: Please rate the importance of the following feelings relative to your use of the concept mapping tool. Do not consider how important these feelings are in your daily life, but please focus specifically on how important they are when you interact with the concept mapping tool. Each of the following sentences includes two ideas that may seem distinct and cause you to hesitate. If this is the case, base your judgment on the feeling that suits you the most.

C3: Please make your assessment now.

For the assessment of the product, please fill out the following questionnaire. The questionnaire consists of pairs of contrasting attributes that may apply to the product. The circles between the attributes represent gradations between the opposites. You can express your agreement with the attributes by ticking the circle that most closely reflects your impression.

Example:

This response would mean that you rate the application as more attractive than unattractive.

Please decide spontaneously. Don’t think too long about your decision to make sure that you convey your original impression. Sometimes you may not be completely sure about your agreement with a particular attribute or you may find that the attribute does not apply completely to the particular product. Nevertheless, please tick a circle in every line. It is your personal opinion that counts. Please remember: there is no wrong or right answer!

Please assess the product now by ticking one circle per line.

C5: Is there any feedback or additional ideas regarding our tool? You are free to describe anything that is important to you.

The next questions are about the topic of the study.

C6: In my opinion, the information about the topic "soil in sustainability" in the learning material is…

C7: I consider the topic "soil in sustainability"…

C8: In my opinion, the visual design of the learning material is…

C9: I consider the visual design of the learning material…

## Footnotes

1 Data from 30 additional participants was collected but excluded from the study because these participants served as a pre-test, used another tool, or were absent in one of the sessions.
